# Forecasting Subjective Cognitive Decline: AI Approach Using Dynamic Bayesian Networks

**DOI:** 10.2196/65028

**Published:** 2025-05-06

**Authors:** Antti Etholén, Teemu Roos, Mirja Hänninen, Ioanna Bouri, Jenni Kulmala, Ossi Rahkonen, Anne Kouvonen, Tea Lallukka

**Affiliations:** 1 Department of Public Health University of Helsinki Helsinki Finland; 2 Department of Computer Science University of Helsinki Helsinki Finland; 3 Western Uusimaa Wellbeing Services County Social and Health Care Services Espoo Finland; 4 Faculty of Social Sciences (Health Sciences) and Gerontology Research Center (GEREC) Tampere University Tampere Finland; 5 Population Health Unit Finnish Institute for Health and Welfare Helsinki Finland; 6 Department of Neurobiology, Care Sciences and Society Division of Clinical Geriatrics, Center for Alzheimer Research Karolinska Institutet Solna Sweden; 7 Faculty of Social Sciences University of Helsinki Helsinki Finland; 8 Centre for Public Health, Queen’s University Belfast Royal Victoria Hospital Belfast United Kingdom

**Keywords:** artificial intelligence, AI, dementia, aging, smoking, alcohol consumption, leisure time physical activity, consumption of fruit and vegetables, body mass index, BMI, insomnia symptoms

## Abstract

**Background:**

Several potentially modifiable risk factors are associated with subjective cognitive decline (SCD). However, developmental patterns of these risk factors have not been used before to forecast later SCD. Practical tools for the prevention of cognitive decline are needed.

**Objective:**

We examined multifactorial trajectories of risk factors and their associations with SCD using an artificial intelligence (AI) approach to build a score calculator that forecasts later SCD. In addition, we aimed to develop a new risk score tool to facilitate personalized risk assessment and intervention planning and to validate SCD against register-based dementia diagnoses and dementia-related medications.

**Methods:**

Five repeated surveys (2000-2022) of the Helsinki Health Study (N=8960; n=7168, 80% women, aged 40-60 years in phase 1) were used to build dynamic Bayesian networks for estimating the odds of SCD. The model structure was developed using expert knowledge and automated techniques, implementing a score-based approach for training dynamic Bayesian networks with the quotient normalized maximum likelihood criterion. The developed model was used to predict SCD (memory, learning, and concentration) based on the history of consumption of fruit and vegetables, smoking, alcohol consumption, leisure time physical activity, BMI, and insomnia symptoms, adjusting for sociodemographic covariates. Model performance was assessed using 5-fold cross-validation to calculate the area under the receiver operating characteristic curve. Bayesian credible intervals were used to quantify uncertainty in model estimates.

**Results:**

Of the participants, 1842 of 5865 (31%) reported a decline in memory, 2818 of 5879 (47.4%) in learning abilities, and 1828 of 5888 (30.7%) in concentration in 2022. Physical activity was the strongest predictor of SCD in a 5-year interval, with an odds ratio of 0.76 (95% Bayesian credible interval 0.59-0.99) for physically active compared to inactive participants. Alcohol consumption showed a U-shaped relationship with SCD. Other risk factors had minor effects. Moreover, our validation confirmed that SCD has prognostic value for diagnosed dementia, with individuals reporting memory decline being over 3 times more likely to have dementia in 2017 (age 57-77 years), and this risk increased to more than 5 times by 2022 (age 62-82 years). The receiver operating characteristic curve analysis further supported the predictive validity of our outcome, with an area under the curve of 0.78 in 2017 and 0.75 in 2022.

**Conclusions:**

A new risk score tool was developed that enables individuals to inspect their risk profiles and explore potential targets for interventions and their estimated contributions to later SCD. Using AI-driven predictive modeling, the tool can aid health care professionals in providing personalized prevention strategies. A dynamic decision heatmap was presented as a communication tool to be used at health care consultations. Our findings suggest that early identification of individuals with SCD could improve targeted intervention strategies for reducing dementia risk. Future research should explore the integration of AI-based risk prediction models into clinical workflows and assess their effectiveness in guiding lifestyle interventions to mitigate SCD and dementia.

## Introduction

Healthy aging is crucial for maintaining individual well-being and managing the capacity of health and social care systems [1]. The economic impact of aging is profound, with health and social care costs surging worldwide [2,3]. In addition, population aging significantly increases the global burden of disease [4]. This situation highlights the urgent need for cost-effective preventive health care strategies. Despite its critical role, preventive health care is often underused, constrained by limited medical consultation times and the prioritization of symptomatic patients [5]. Therefore, there is a pressing need for innovative, efficient, and cost-effective strategies to enhance preventive measures. Early identification of risky lifestyles already in midlife is essential; it can delay the onset of health deterioration in later years and significantly improve the quality of life by adding healthy years to life [6].

Cognitive functioning is a crucial factor for functional ability and well-being in later life [7]. Subjective cognitive decline (SCD)—self-perceived worsening of cognitive abilities, such as memory, learning, or concentration, despite normal performance on objective cognitive tests—is increasingly recognized as one of the potential early warning signs of dementia [8,9]. Risk factors for cognitive decline include high age, the ApoE4 genotype, cardiovascular and metabolic conditions, mental health issues, low education, and unhealthy lifestyle factors [7,10]. Since the underlying cause of cognitive decline is still not fully understood, there are currently no curative treatments, and thus, early detection and management of their risk factors are critical [11]. Although traditional cognitive assessments remain valuable, innovative approaches are emerging to both assess and intervene in cognitive decline. Recent work has demonstrated the efficacy of technology-based interventions, such as virtual reality, for enhancing cognitive functioning in those already experiencing mild cognitive impairment [12].

Traditional predictive models, such as logistic regression and Cox proportional hazards models, often assume linear relationships and may struggle to capture complex, time-dependent risk interactions. Microsimulation models simulate individual trajectories but rely on predefined transition structures, which may limit flexibility. In contrast, dynamic Bayesian networks (DBNs) offer a data-driven approach that models probabilistic dependencies over time, providing greater adaptability in uncovering latent patterns and health trajectories. DBNs allow for modeling complex, nonlinear relationships among variables and handling missing data efficiently. Building on these advantages, this study applied advanced artificial intelligence (AI) techniques, particularly DBNs, to robustly analyze complex data, reveal latent patterns over time, and identify risk-related patterns for SCD. This approach promises a more personalized and proactive identification of health trajectories [13-15].

The main aim of this study was to produce new information about multifactorial trajectories of risk factors and their associations with SCD using an AI approach to develop a new score tool to forecast SCD. Furthermore, the aim was to validate an SCD instrument with objective register data on dementia. The main aim was divided into 4 objectives:

- Developing and applying AI models: This objective included using advanced AI technologies to develop and assess a DBN for risk estimation and modeling risk factor trajectories. A key element was the creation of the Helsinki Health Study (HHS) score for predicting SCD using indicators such as smoking, alcohol consumption, leisure time physical activity (LTPA), consumption of fruit and vegetables, BMI, and insomnia symptoms, adjusting for sociodemographic factors.
- Using the HHS score to predict SCD: This objective focused on providing examples of how the HHS score can help individuals assess their health risks and make informed decisions.
- Creating a new tool: The final objective was to build an interactive online calculator that allows users to enter their risk factors and estimate changes in SCD over 5 years based on chosen intervention.
- Validating SCD against objective register-based data on dementia diagnoses and dementia medications for the first time: This objective helped confirm the results and provided new information about the reliability and validity of survey-based outcomes for future use.

## Methods

### Data

**Figure 1:**
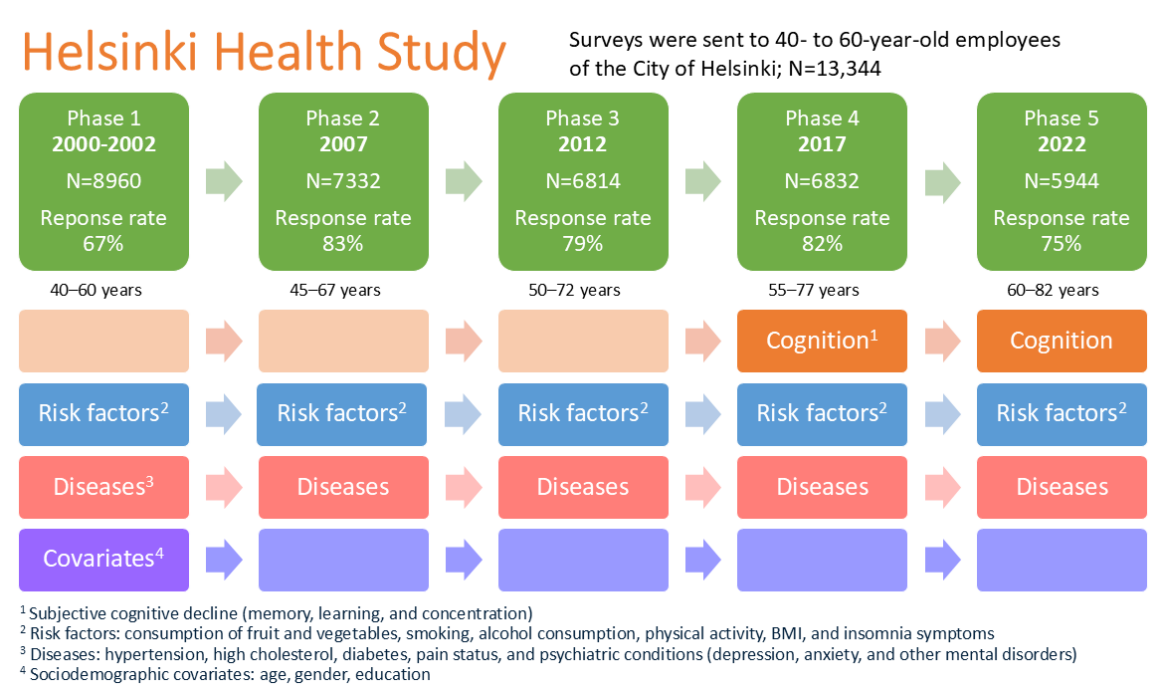
Phases of the cohort: outcome (SCD) and exposure (risk factors) used in different study phases. HHS: Helsinki Health Study; SCD: subjective cognitive decline.

Data for this study were obtained from the HHS (Figure 1) [16]. The initial survey was conducted between 2000 and 2002, targeting 40- to 60-year-old employees of the City of Helsinki, Finland (phase 1, N=8960, response rate 67%). Follow-up surveys were administered to all phase 1 participants in 2007 (phase 2, response rate 83%), 2012 (phase 3, response rate 79%), 2017 (phase 4, response rate 82%), and 2022 (phase 5, response rate 75%). The final analytic sample comprised 8960 individuals.

### Measurements

#### Risk Factors

Risk factors [7,17] were categorized following the current national guidelines [18] and were used as time-dependent variables: consumption of fruit and vegetables (daily consumption of both, daily consumption of either, nondaily consumption), smoking (never smoked, ex-smoker, and smoker), alcohol consumption (no consumption; moderate consumption: 1-13 units of alcohol at least once a week among men and 1-6 units among women; high consumption: at least 14 units among men and 7 units among women; very high-risk consumption: at least 23 units among men and 12 units among women), LTPA (physically inactive: metabolic equivalent (MET)<14; physically active: MET≥14), BMI (underweight: <18.5; recommended healthy weight: 18.5-24.9; overweight: 25.0-29.9; obesity: ≥30) [19], and insomnia symptoms (<4, 4-14, >14 nights/month).

#### Subjective Cognitive Decline

Three variables were used to measure SCD. Participants were asked to report “How well my memory works,” “How well embracing and learning new things goes for me,” and “Normally I can concentrate on something” [17,20]. The instrument is included in the TOIMIA (The Functioning Measures) database, which is maintained by the Finnish Institute for Health and Welfare [21]. The items were used to define 3 different aspects of self-rated cognitive functioning: memory, learning, and concentration. Participants rated themselves on a 5-point scale (“very poorly,” “poorly,” “satisfactorily,” “well,” and “very well”). The scales were recategorized as good (including “well” and “very well”) and declined (including “satisfactorily,” “very poorly,” and “poorly”). The first register-based validation of the measure is available in Multimedia Appendix 1.

#### Sociodemographic Covariates

Age was used as a dynamic (time-dependent) variable and segmented into the closest age groups (40-80 years with 5-year intervals). Additionally, other variables served as static variables in the DBN model: gender (women, men; phase 1) and education (basic, secondary, higher; phase 1).

#### Health Factors and Physician-Diagnosed Diseases

Several health factors were included to underscore their potential associations with SCD. These factors encompassed the current pain status (no, yes) and the following physician-diagnosed conditions: hypertension, high serum cholesterol, diabetes, and mental disorders (depression, anxiety disorder, other mental disorder).

### Statistical Analysis

#### AI Approach in the Development of the HHS Score

We developed the HHS score using DBNs, allowing detailed analysis of the relationships among covariates, variables, and outcomes over time segments [22]. DBNs, structured as directed acyclic graphs, include nodes representing variables and edges denoting probabilistic dependencies [23]. This structure offers a transparent analytical framework, enhancing model predictability and accuracy. A time-homogeneous DBN maintains consistency, while allowing influence from previous time steps. We modeled temporal progression by age, with each step representing 5 years, accurately depicting health-related variable progression.

Statistical analyses and model building were performed using the C# programming language [24] with the R.NET library [25] functioning as an in-process bridge to integrate R version 4.2.1 (R Foundation for Statistical Computing) [26] with the *bnlearn* package [27]. Probabilistic inferences within the network were facilitated using the *ML.NET* probabilistic package [28].

### Data Preparation

Outliers were treated as missing values. We generated descriptive contingency tables to illustrate missing data and the sizes of each variable group across phases 1-5. Due to the infrequency of missing covariate data and the capability of Bayesian networks to handle such values, we did not perform any imputations [29]. Moreover, the full dataset could be used in the model, as each node’s conditional probability table (CPT) was estimated based on the available cases for that specific variable, without requiring complete data across all variables. All categorical variables were treated as discrete random variables, with domains that were mutually exclusive and exhaustive.

### Implementing the DBN Model

Initially, we established the model structure using expert knowledge and automated techniques. Temporal variables (age group, consumption of fruit and vegetables, smoking, alcohol consumption, LTPA, BMI, and insomnia symptoms) were hardwired between time slices to represent development paths. We then used a score-based quotient normalized maximum likelihood (qNML) criterion algorithm to identify additional relationships within the same time slice (horizontal effects) between nodes. qNML is an information-theoretic model selection criterion similar to the commonly used Bayesian information criterion (BIC) [30]. It identifies the most informative parent sets for each variable, while penalizing model complexity. qNML has been found to favor models that, although parsimonious, tend to achieve higher predictive performance than models selected by BIC for moderate and large sample sizes [31]. In the qNML algorithm, the nodes were ordered as follows: age group, gender, education, consumption of fruit and vegetables, smoking, alcohol consumption, LTPA, insomnia symptoms, BMI, hypertension, high cholesterol, diabetes, psychiatric disorders, current pain status, concentration, memory, and learning. The decomposable qNML algorithm facilitates separate structural learning for each node and its potential parents. To manage complexity, we limited each node to a maximum of 3 parents. Variables were organized so that causative variables preceded effects, but constructing a causal network was not our intent [32]. We assessed combinations of up to 3 parents for each node, selecting the configuration with the highest qNML score. Next, we derived CPTs for each node using our complete dataset. To address the zero-cell problem in probability estimation, we applied the Bayesian smoothing algorithm with a Dirichlet prior (1/2,...,1/2) to each CPT.

### Inference Using the Model

The use of a Bayesian network offers significant advantages, primarily the ability to perform inference based on specified evidence and events. Age is the initial parameter in this dynamic model. For the initial time slice, CPTs represented prior probabilities from our dataset. This inference mechanism supports a scoring calculator, where users input specific data into the network. This allows the calculation of event probabilities, such as the likelihood of memory decline or probabilities related to the longitudinal development of variables. Here, “evidence” referred to user-provided data used to infer the HHS score based on a pretrained DBN.

The expectation propagation algorithm, a deterministic method, was used for inference, yielding approximate results in discrete cases [33]. In inference, information contained in the evidence provided by the user is propagated through the network one variable at a time, updating the probability distribution of each variable by conditioning on the evidence. We calculated the full posterior distribution for each variable within each time slice (t) to maintain uncertainty and probabilistic relationships among variables. Results were presented with Bayesian credible intervals, which inherently account for uncertainty and ensure robust estimation of relationships, while preserving probabilistic dependencies over time. When transitioning from one time slice to the next, we used the Maximum A Posteriori (MAP) estimates of the variables from the previous time slice (t – 1) as inputs for calculating the current time slice (t) to reduce computational complexity and prevent the overpropagation of uncertainty. Cross-correlations and autocorrelations among output variables were also calculated from our dataset, based on demographic and risk factors.

### Evaluation Metrics

We validated our model using 5-fold cross-validation and evaluated classification performance by calculating the area under the receiver operating characteristic curve (AUROC) [34].

### Decision Heatmap

We introduced a dynamic decision heatmap as a communication tool, for instance, in patient consultations. This heatmap displayed the current state of a patient’s lifestyle and presented a decision matrix for each modifiable risk factor. It illustrated the effects of potential changes, projected 5 years into the future, showing risk changes derived from the DBN. When selecting a target intervention, the heatmap initiated precalculation in the DBN using the chosen intervention as evidence, generating new estimates for SCD to facilitate informed decision-making.

### Ethical Considerations

This study was approved and received a research permit from the health authorities of the City of Helsinki. The study plan was approved by the Ethical Committee of the Faculty of Medicine, University of Helsinki, Finland. Permission for the secondary use of health and social data was granted by the Finnish Social and Health Data Permit Authority, Findata. Participants provided informed consent for scientific research. All data were handled in compliance with data protection regulations.

## Results

### Participant Details

The study population was based on survey data from the HHS (2000-2022). The baseline (phase 1) study population consisted of 40- to 60-year-old employees of the City of Helsinki, Finland (Table 1). Multimedia Appendix 2 shows the response rates by variable and study phase. Of all participants, 1842 (31%) reported a decline in memory, 2818 (47.4%) in learning abilities, and 1828 (30.7%) in concentration in 2022 (Table 2). Nondaily consumption of fruit and vegetables was reported by 892 (15.4%) to 2120 (23.9%) of the participants, while 3346 (48.2%) participants in phase 2 and 3094 (53%) participants in phase 4 were current or former smokers. Additionally, 575 (10.1%) to 1339 (19.0%) of the participants engaged in high or high-risk alcohol consumption. Physical inactivity was reported by 1650 (22.7%) to 1776 (26.5%) of the participants. At phase 5, 2183 (37.2%) had overweight, and 1312 (22.4%) had obesity. Finally, at phase 5, insomnia symptoms occurring >14 nights per month were reported by 1431 (27.2%) of the participants (Table 3). Furthermore, in phase 5 a total of 2529 (44.3%) of the participants reported pain, 2999 (59.8%) reported hypertension, 2608 (56.2%) reported high cholesterol levels, 837 (20.6%) reported diabetes, and 827 (20.4%) reported mental disorders (Table 4).

**Table 1:** Sociodemographic characteristics and DBN^a^ node names.

Characteristics			Phases								
			Phase 1 (2000-2002; N=8960), n (%)		Phase 2 (2007; N=7332), n (%)		Phase 3 (2012; N=6808), n (%)		Phase 4 (2017; N=6831), n (%)		Phase 5 (2022; N=5950), n (%)
**Gender**											
	Men	1792 (20.0)		—^b^		—		—		—	
	Women	7168 (80.0)		—		—		—		—	
**Age (years)**											
	40	1800 (20.1)		—		—		—		—	
	45	1902 (21.2)		1401 (19.1)		—		—		—	
	50	1944 (21.7)		1497 (20.4)		1245 (18.3)		—		—	
	55	2230 (24.9)		1594 (21.7)		1393 (20.5)		1319 (19.3)		—	
	60	1084 (12.1)		1907 (26.0)		1496 (22.0)		1461 (21.4)		1213 (20.4)	
	65	—		933 (12.7)		1811 (26.6)		1518 (22.2)		1350 (22.7)	
	70	—		—		863 (12.7)		1744 (25.5)		1323 (22.2)	
	75	—		—		—		789 (11.6)		1449 (24.4)	
	80	—		—		—		—		615 (10.3)	
**Education**											
	Basic	3790 (42.3)		—		—		—		—	
	Secondary	2793 (31.2)		—		—		—		—	
	Higher	2298 (25.6)		—		—		—		—	
	No answer	79 (0.9)		—		—		—		—	

^a^DBN: dynamic Bayesian network.

^b^Not applicable.

**Table 2:** SCD^a^ symptoms and DBN^b^ node names.

Symptoms and nodes		Phases^c^								
		Phase 1 (2000-2002), n/N (%)		Phase 2 (2007), n/N (%)		Phase 3 (2012), n/N (%)		Phase 4 (2017), n/N (%)		Phase 5 (2022), n/N (%)
**Memory**										
	Good	—^d^	—		—		4568/6762 (67.6)		4023/5865 (68.6)	
	Declined	—	—		—		2194/6762 (32.4)		1842/5865 (31.4)	
**Learning**										
	Good	—	—		—		3513/6714 (52.3)		3061/5879 (52.1)	
	Declined	—	—		—		3201/6714 (47.7)		2818/5879 (47.9)	
**Concentration**										
	Good	—	—		—		4668/6718 (69.5)		4060/5888 (69.0)	
	Declined	—	—		—		2050/6718 (30.5)		1828/5888 (31.0)	

^a^SCD: subjective cognitive decline.

^b^DBN: dynamic Bayesian network.

^c^The number of participants used includes only those who responded to the surveys.

^d^Not applicable.

**Table 3:** Risk factors and DBN^a^ node names.

Factors and nodes		Phases^b^				
		Phase 1 (2000-2002), n/N (%)	Phase 2 (2007), n/N (%)	Phase 3 (2012), n/N (%)	Phase 4 (2017), n/N (%)	Phase 5 (2022), n/N (%)
**Smoking**						
	Never	4336/8518 (50.9)	3593/6939 (51.8)	3080/6116 (50.4)	2744/5838 (47.0)	2361/5016 (47.1)
	Ex-smoker	2066/8518 (24.3)	2033/6939 (29.3)	2085/6116 (34.1)	2277/5838 (39.0)	2146/5016 (42.8)
	Current smoker	2116/8518 (24.8)	1313/6939 (18.9)	951/6116 (15.5)	817/5838 (14.0)	509/5016 (10.1)
**Alcohol consumption**						
	No consumption	604/8573 (7.0)	598/7067 (8.5)	685/6548 (10.5)	943/6625 (14.2)	1038/5671 (18.3)
	Moderate consumption	6558/8573 (76.5)	5130/7067 (72.6)	4813/6548 (73.5)	4761/6625 (71.9)	4058/5671 (71.6)
	High consumption	835/8573 (9.7)	720/7067 (10.2)	584/6548 (8.9)	531/6625 (8.0)	323/5671 (5.7)
	High-risk consumption	576/8573 (6.7)	619/7067 (8.8)	466/6548 (7.1)	390/6625 (5.9)	252/5671 (4.4)
**LTPA^c^**						
	Physically inactive (MET^d^<14)	2237/8869 (25.2)	1650/7256 (22.7)	1776/6711 (26.5)	1628/6719 (24.2)	1444/5896 (24.5)
	Physically active (MET>14)	6632/8869 (74.8)	5606/7256 (77.3)	4935/6711 (73.5)	5091/6719 (75.8)	4452/5896 (75.5)
**Fruit and vegetable consumption**						
	Nondaily consumer	2120/8873 (23.9)	1438/7203 (20.0)	1255/6675 (18.8)	1438/6635 (21.6)	892/5811 (15.4)
	Daily consumer of either	2596/8873 (29.3)	2038/7203 (28.3)	1820/6675 (27.3)	1662/6635 (25.0)	1396/5811 (24.0)
	Daily consumer of both	4157/8873 (46.8)	3727/7203 (51.7)	3600/6675 (53.9)	3558/6635 (53.4)	3523/5811 (60.6)
**BMI**						
	Underweight (<18.5) or recommended healthy weight (18.5-24.9)	4551/8853 (51.4)	3343/7252 (46.1)	2843/6706 (42.4)	2721/6743 (40.4)	2368/5863 (40.4)
	Overweight (25.0-29.9)	3027/8853 (34.2)	2588/7252 (35.7)	2461/6706 (36.7)	2548/6743 (37.8)	2183/5863 (37.2)
	Obesity (≥30.0)	1275/8853 (14.4)	1321/7252 (18.2)	1402/6706 (20.9)	1474/6743 (21.9)	1312/5863 (22.4)
**Insomnia symptoms (nights/month)**						
	<4	4032/8216 (49.1)	2870/6719 (42.7)	2733/6189 (44.2)	2499/5855 (42.7)	2228/5260 (42.4)
	4-14	2596/8216 (31.6)	2166/6719 (32.2)	1912/6189 (30.9)	1749/5855 (29.9)	1601/5260 (30.4)
	>14	1588/8216 (19.3)	1683/6719 (25.0)	1544/6189 (24.9)	1607/5855 (27.4)	1431/5260 (27.2)

^a^DBN: dynamic Bayesian network.

^b^The number of participants used includes only those who responded to the surveys.

^c^LTPA: leisure time physical activity.

^d^MET: metabolic equivalent.

**Table 4:** Physician-diagnosed diseases and DBN^a^ node names.

Physician-diagnosed diseases and nodes		Phases^b^				
		Phase 1 (2000-2002), n/N (%)	Phase 2 (2007), n/N (%)	Phase 3 (2012), n/N (%)	Phase 4 (2017), n/N (%)	Phase 5 (2022), n/N (%)
**Pain**						
	No	4852/8755 (55.4)	3928/7106 (55.3)	3838/6648 (57.7)	3653/6494 (56.3)	3182/5711 (55.7)
	Yes	3903/8755 (44.6)	3178/7106 (44.7)	2810/6648 (42.3)	2841/6494 (43.7)	2529/5711 (44.3)
**Hypertension**						
	No	6834/8865 (77.1)	4834/7207 (67.1)	4115/6699 (61.4)	2632/5431 (48.5)	2018/5017 (40.2)
	Yes	2031/8865 (22.9)	2373/7207 (32.9)	2584/6699 (38.6)	2799/5431 (51.5)	2999/5017 (59.8)
**High cholesterol**						
	No	6989/8874 (78.8)	4725/7252 (65.2)	3847/6773 (56.8)	2734/5067 (54.0)	2032/4640 (43.8)
	Yes	1885/8874 (21.2)	2527/7252 (34.8)	2926/6773 (43.2)	2333/5067 (46.0)	2608/4640 (56.2)
**Diabetes**						
	No	7652/7889 (97.0)	5291/5680 (93.2)	4737/5344 (88.6)	3855/4658 (82.8)	3224/4061 (79.4)
	Yes	237/7889 (3.0)	389/5680 (6.8)	607/5344 (11.4)	803/4658 (17.2)	837/4061 (20.6)
**Mental disorders**						
	No	6600/8102 (81.5)	4547/5897 (77.1)	4297/5443 (78.9)	3683/4638 (79.4)	3229/4056 (79.6)
	Yes	1502/8102 (18.5)	1350/5897 (22.9)	1146/5443 (21.1)	955/4638 (20.6)	827/4056 (20.4)

^a^DBN: dynamic Bayesian network.

^b^The number of participants used includes only those who responded to the surveys.

### DBN Model Implementation

**Figure 2:**
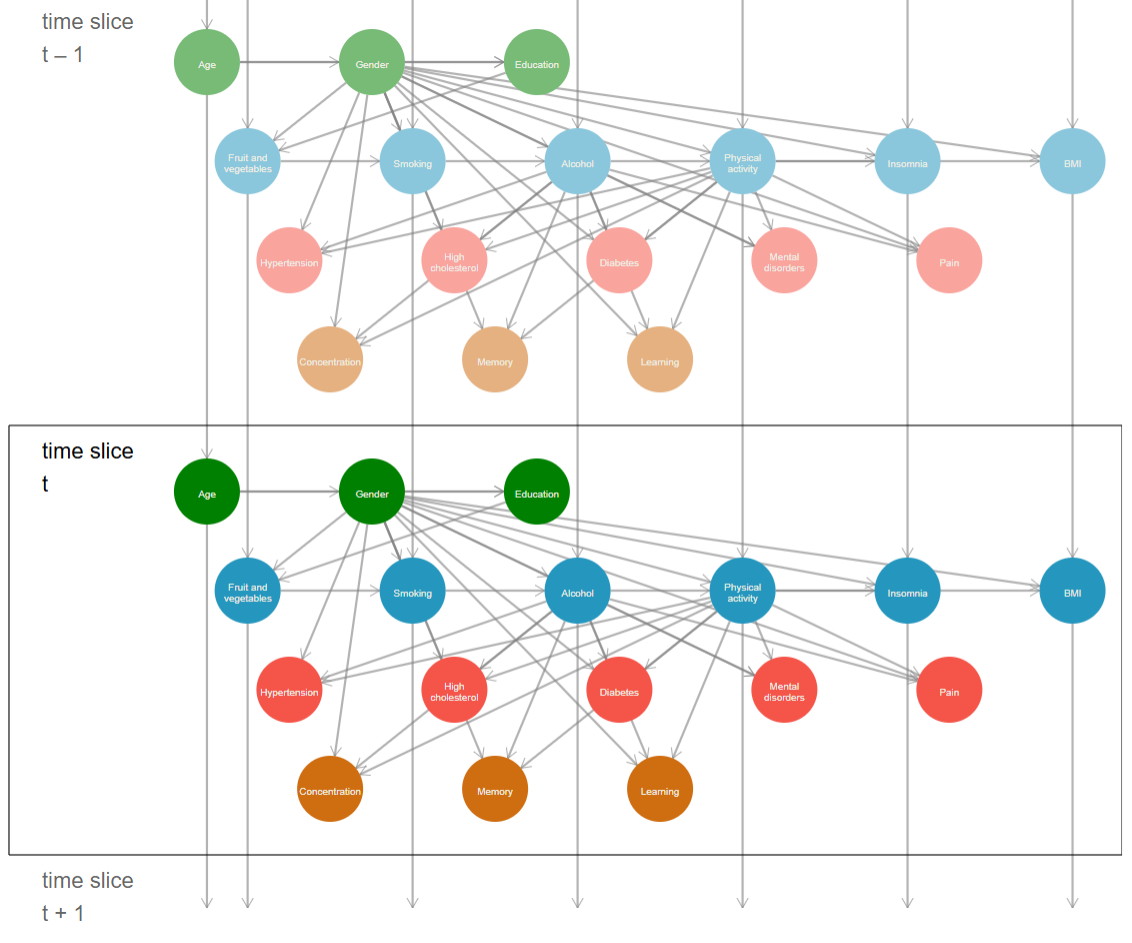
HHS score DBN model for predicting SCD based on expert knowledge and the score-based qNML criterion algorithm. The flow (possible parents of the current node) of the network is from left to right and from top to bottom. Definitions for nodes: age=age group (5-year intervals), gender (woman, man), education (basic, secondary, higher), fruit and vegetables=consumption of fruit and vegetables (daily consumption of both, daily consumption of either, nondaily consumption), smoking (never smoked, ex-smoker, and smoker), physical activity=LTPA (physically inactive: MET<14; physically active: MET≥14), BMI (underweight: <18.5; recommend healthy weight 18.5-24.9; overweight: 25.0-29.9; obesity: ≥30), insomnia (<4, 4-14, >14 nights/month), hypertension (no, yes), highcholestrol=high cholesterol (no, yes), diabetes (no, yes), mental disorders (no, yes; including depression, anxiety disorder, other mental disorder), pain=current pain status (no, yes), concentration (good, declined), memory (good, declined), learning (good, declined). Total training size N=8960 based on 5 repeated surveys (2000-2022) of the HHS. DBN: dynamic Bayesian network; HHS: Helsinki Health Study; LTPA: leisure time physical activity; MET: metabolic equivalent; qNML: quotient normalized maximum likelihood; SCD: subjective cognitive decline.

The structure of the DBN model, trained using qNML, is illustrated in Figure 2. The most informative parent nodes for the nodes representing memory, learning, and concentration were gender, alcohol consumption, and LTPA. These same parent nodes were selected for the nodes representing pain, hypertension, high cholesterol, diabetes, and mental disorders.

Analysis of the CPTs presented in Multimedia Appendix 3 indicated that LTPA consistently reduces the probability of SCD. Specifically, the average relative risk reduction of experiencing declined memory when transitioning from inactive to active physical activity states was 26.9% for men and 24.8% for women across different levels of alcohol consumption. Additionally, the relationship between alcohol consumption and SCD followed a U-shaped curve, with both nonconsumption and high-risk consumption exerting greater effects than moderate or elevated consumption. Manual testing of the network, corroborated by data from our sampled participants, supported these findings.

### Decision Heatmap

**Figure 3:**
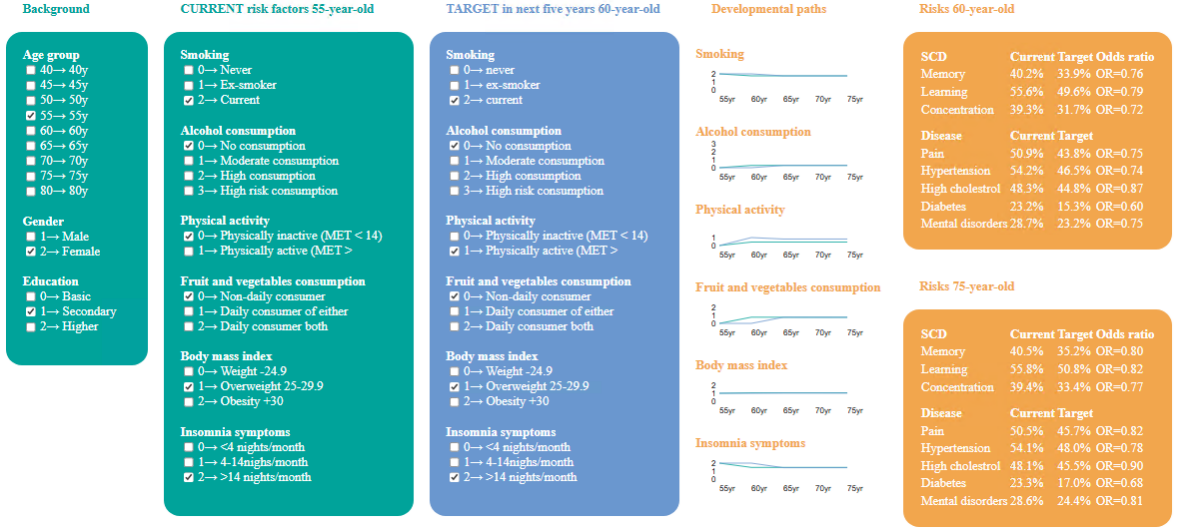
HHS score tool user interface allows users to input their current risk factors, demographic variables, and potential targets for 5 years later, generating trajectories of risk factors and risks associated with SCD and health factors. For example, in this case, the current evidence is “55 years, woman, physically inactive” and the target evidence is “55 years, woman, physically inactive (55 years), physically active (60 years).” The risk of declined memory when 60 years old based on current evidence is 40.1% and based on target evidence is 33.9%, having an OR of 0.76 (95% Bayesian credible interval 0.59-0.99). HHS: Helsinki Health Study; MET: metabolic equivalent; OR: odds ratio; SCD: subjective cognitive decline.

The user interface of the HHS score calculator is presented in Figure 3. The inputs for the calculator were set as evidence for Bayesian inference. The probabilities for each output, including developmental paths and risks, were read from the inferred model.

**Figure 4:**
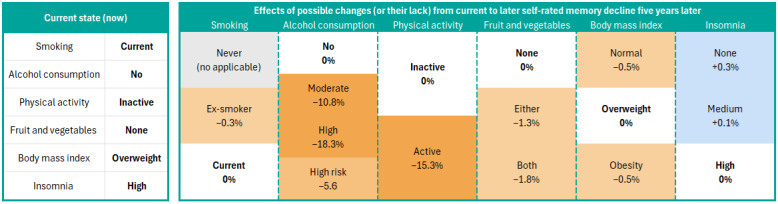
The first step in the decision heatmap to decrease self-reported memory decline in a 55-year-old woman with a secondary education. The decision matrix shows the effects of possible changes 5 years from now based on the current state. When the first choice is made, the evidence changes, and the new values to the matrix are updated, making this decision heatmap dynamic.

We selected an individual example from our sample to demonstrate the use of the HHS score calculator (Figure 3). This individual was a 55-year-old woman with secondary education who did not eat fruit and vegetables daily, currently smoked, did not consume alcohol, was physically inactive, with overweight, and had high insomnia symptoms. In a consultation with a health care professional, the decision heatmap (Figure 4) was used to communicate with the individual and propose personalized lifestyle modifications. For instance, a strategy focused on increasing LTPA over the next 5 years appears to be particularly effective. As shown in Figure 3, the outcomes indicated that, for example, the likelihood of subjective memory decline decreased from 40.2% to 33.9%, with an absolute likelihood reduction of 6.3 percentage points and an odds ratio (OR) of 0.76 (95% Bayesian credible interval 0.59-0.99). Considering that continuing the current lifestyle was estimated to lead to a 49.2% likelihood of memory decline, an intervention resulting in a reduction of 15.3 percentage points was estimated to decrease this likelihood to 33.9% (Figure 4).

We further found statistically significant cross-correlations between a reduction in SCD and various commonly reported health conditions, including hypertension (*P*<.001), high serum cholesterol (*P*<.001), and diabetes (*P*<.001), based on a sample at the initial time step, t (Multimedia Appendix 4). Mental disorders and pain showed minor contributions to the reduction in SCD. Following the intervention, the effects intensified at time step (t + 1), and subsequent autocorrelation analysis indicated stabilization from (t + 1) to (t + 3).

### Model Performance Cross-Validation

The model’s predictive power measured by the area under the curve (AUC) metric was moderate; the cross-validated AUC scores were in the range of 0.52-0.65, as shown in Multimedia Appendix 5. This indicated that the model provides meaningful estimates of average risk differences across lifestyle profiles.

## Discussion

### Principal Findings

We demonstrated how an AI approach with DBNs can be used to build a risk score for forecasting SCD, effectively handling complex interactions between different factors, and providing clear, interpretable outcomes. We found that LTPA appears to have the most consistent associations with subsequent SCD. In addition, we discovered that SCD and disease risks, such as diabetes, hypertension, and high serum cholesterol, are highly correlated.

### Interpretation

The nature of SCD is multifactorial and may arise from normal aging (as older adults are more likely to monitor their memory and raise concerns), be reversible, or progressively lead to dementia [8]. SCD is prevalent in aging populations, and prevention of dementia is a global priority for the World Health Organization (WHO) [35,36]. Additionally, a lifestyle intervention program has been found to have beneficial effects for preventing cognitive decline in at-risk older adults [37]. This highlights the importance of the preventive HHS score, which addresses multifactorial issues through advanced AI-based DBNs to analyze complex data and identify emerging risk groups already in midlife.

Our study shows the complex associations between SCD and alcohol use, smoking, LTPA, consumption of fruit and vegetables, BMI, and insomnia. We found that changing these potentially modifiable factors could decrease the overall likelihood of SCD. Notably, LTPA emerged as the most significant predictor in our model, decreasing the risk of SCD. Numerous interventions have aimed to enhance cognitive functioning. For example, a systematic review by Barha et al [38] showed that, especially, aerobic exercise has a positive impact on cognition (standardized mean difference 0.85, SD higher compared to controls) [38].This effect size aligns with previous cohort studies, where high physical activity in mid-to-late life has been associated with up to a 38% lower risk of SCD [39] and physical inactivity has been linked to increased odds of SCD [40]. This finding aligns with the well-documented benefits of LTPA on cognitive function [41,42]. Other studies also corroborate that physical inactivity is a significant risk factor for SCD [43,44]. Many studies demonstrate the preventive importance of physical activity in achieving healthy aging and preventing frailty [45]. Increased physical activity can improve cerebral blood flow, enhance synaptic plasticity, facilitate cellular cleanup processes, decrease beta-amyloid levels, improve mental health, and reduce stress [46]. The novelty of this study lies in a new tool that assesses how changes in risk factors impact SCD during follow-up and how increased physical activity, for example, can reduce its likelihood.

The observed correlation between SCD and other disease risks may indicate shared underlying mechanisms or common risk factors. This suggests that interventions targeting cardiovascular risk factors could concurrently improve SCD [47]*.* Cognitive decline is also influenced by genetic factors, such as the ApoE4 genotype, as well as health conditions, which may interact with modifiable lifestyle factors [7]. Future studies should explore these interactions to better understand their role in the risk for SCD.

### Practical Applications

Given the shared risk factors, insights into SCD can provide valuable indications about potential progression to dementia. However, since our findings are based on observational data, the projected associations illustrated by the decision heatmap and the HHS score calculator should not be interpreted as direct evidence of intervention efficacy without confirmatory intervention studies. Although the longitudinal design, in which risk factors were assessed prior to the onset of SCD, supports the possibility of a causal relationship, it does not confirm that modifying these risk factors would influence the outcome. For that, randomized controlled trials (RCTs) are needed. However, the follow-up time in RCTs is often short, and the long-terms effects of interventions are difficult to capture, particularly for health outcomes that develop slowly (eg, dementia). Furthermore, it is possible to produce findings like RCTs even with observational data [48]. Prior to broader implementation, it is crucial to conduct an intervention study focusing on the identified risk factors to determine whether risk factor modifications can, indeed, lower the risk for SCD or dementia in line with the results suggested by the algorithm and what the adherence is to different lifestyle interventions in the long run.

The HHS score partially overlaps with existing dementia risk scores in terms of included lifestyle-related predictors. For example, the CAIDE (Cardiovascular Risk Factors, Aging, and Dementia) score incorporates the BMI and physical inactivity but not smoking, alcohol, diet, or sleep-related factors [49], while the LIBRA (Lifestyle for Brain Health) score covers most lifestyle components—smoking, alcohol, diet, and physical activity—but does not include insomnia [50]. Unlike these additive risk models estimating long-term dementia risk, the HHS score uses a DBN to predict SCD based on longitudinal data and interdependencies between variables. This approach allows for more individualized, time-sensitive predictions. A recent review emphasized the need for more dynamic, personalized tools for cognitive risk estimation, particularly those accounting for time-varying exposures [50].

In clinical settings, our score calculator could assist in assessing the probabilities of SCD. Additionally, our decision heatmap can support health care professionals when discussing how lifestyle changes could impact health outcomes with their patients. Our methodology integrates decision theory—combining elements of probability theory and utility theory—to evaluate the effectiveness of preventive measures [51]. For example, our DBN identified a U-shaped curve in alcohol consumption, similar to a large meta-analysis [52]. This finding exemplifies how latent effects can influence risk assessments and should be interpreted with respect to probabilities. This integrated approach ensures that recommendations are personalized and rooted in a comprehensive assessment of each patient’s unique health profile and needs. To support real-world clinical use, potential implementation barriers, such as integration with electronic health records, time constraints during appointments, and provider training, should also be considered. However, given the moderate predictive power (AUC 0.52-0.65), the HHS score should currently be considered a supportive tool rather than a definitive diagnostic instrument. Further external validation and refinement are needed before clinical implementation.

### Limitations and Strengths

Our study has a few limitations. First, our attrition analysis [17] indicated that individuals with SCD are more likely to drop out in subsequent phases. Second, our results cannot be directly generalized to the general population, as the cohort was female dominated and comprised individuals who were employees of the City of Helsinki at inclusion. Although this homogeneity reduces confounding, it may limit external validity. The HHS score may perform differently in populations with more diverse socioeconomic backgrounds, in the general population, among people with different occupations and health care access, or in male-dominated populations. Importantly, gender was identified as an informative parent node for several other variables in the model. This means that due to the underrepresentation of men in the cohort, the CPTs for male-specific paths in the network were estimated from fewer observed values, potentially affecting the model’s performance and stability in male-dominated or gender-balanced populations. Future studies should validate the model in more heterogeneous samples.

Despite these limitations, our study also has significant strengths. First, our >20-year longitudinal cohort used identical questionnaires across all 5 phases, achieving moderate-to-excellent response rates for a broad range of sociodemographic and health-related variables. Nonresponse analyses confirmed that the data accurately represented the target population [16]. Second, we used an AI approach to better account for the complex dynamics and interactions among our variables. A DBN is an ideal model for our calculator because of its ability to process various types of data. It can effectively manage cases where some nodes are in an undefined state, labeled as “NA,” without compromising its functionality. This model simplifies the complexity typically encountered in networks by avoiding connections between every pair of variables. Instead, it links variables only across consecutive time slices. Unlike deep learning, which often requires large datasets and can suffer from reduced interpretability, DBNs are well suited for modeling structured relationships in smaller datasets. Additionally, DBNs allow for the simultaneous analysis of dynamic interactions among multiple variables over time, rather than assuming linear relationships as in traditional regression models. Additionally, the DBN model is characterized by its transparency, allowing users to inspect and understand internal processes. Moreover, it can handle incomplete datasets without requiring data imputation to fill missing values. However, DBNs are not without limitations. They can be sensitive to data quality, particularly when dealing with small sample sizes or noisy data, and they may be prone to overfitting if not properly regularized. Third, a key strength of the study is that we were able to conduct a first validation of the SCD against objective register data on dementia (medication data and hospitalizations). Our validation demonstrated a strong association between SCD and clinically verified dementia diagnoses, with individuals reporting memory decline being over 3 times more likely to have dementia in 2017 (age 57-77 years), and this risk increased to more than 5 times by 2022 (age 62-82 years). Additionally, declines in learning and concentration showed similar trends, reinforcing the role of SCD as an early indicator of later dementia risk. The receiver operating characteristic curve (ROC) analysis further confirmed its predictive validity, with an AUC of 0.78 in 2017 and 0.75 in 2022. Our measurement of SCD had good internal consistency, and consistency remained between phase 4 (2017) and phase 5 (2022). The consistent trends observed across phases suggest that the measure reliably captures the associations between SCD and many health-related factors and dimensions of health functioning. These findings confirm that SCD is a valid and reliable outcome measure, with significant prognostic value for dementia (Multimedia Appendix 1). This helps confirm our results and supports the future use of survey-based SCD when examining cognitive functioning and its risk factors.

### Conclusion

At the individual level, the HHS score can provide concrete examples and motivation for understanding one’s future potential developmental patterns of risk factors and the contributions of their changes to later SCD. It could be used to detect the risk of adverse development early, especially when lifestyle information is included in the risk assessment by the patient or a health professional. Early detection and interventions already in midlife can improve the quality of life and add healthier years to life. From a broader perspective, these findings emphasize the need for AI-driven tools in public health and health care policy. Predictive models, such as the HHS score, could be integrated into preventive health care initiatives, supporting policymakers in designing evidence-based interventions to reduce the burden of cognitive decline. By using personalized health assessments, health care systems can proactively address modifiable risk factors and improve long-term cognitive outcomes in aging populations.

Future research should assess whether long-term lifestyle interventions can reduce dementia and SCD risk, as demonstrated in FINGER (Finnish Intervention Study to Prevent Cognitive Decline and Disability) [53]. However, showing their effectiveness in preventing clinically diagnosed dementia remains challenging. AI-driven tools could help by modeling long-term intervention effects, integrating biomarkers to improve risk prediction, and supporting personalized prevention strategies. Additionally, the HHS score should be validated in more diverse populations, and other potentially modifiable risk factors of SCD should be further explored.

## Appendix

Multimedia Appendix 1 Validation of the subjective cognitive decline (SCD) instrument.

Multimedia Appendix 2 Response rates by variable and study phase.

Multimedia Appendix 3 Conditional probability tables (CPTs) of subjective cognitive decline (SCD) and leisure time physical activity (LTPA) in dynamic Bayesian networks (DBNs).

Multimedia Appendix 4 Spearman rank cross-correlations and autocorrelations between subjective cognitive decline (SCD) and additional outcome variables in a sample of 35,186 individuals, based on model inference using phased data from our original dataset. Autocorrelations between time slices, t=0 and t=1, are presented on the diagonal, while cross-correlations within t=0 are presented off-diagonal.

Multimedia Appendix 5 Area under the receiver operating characteristic curve (AUROC) calculated using 5-fold cross-validation for the outcomes of subjective cognitive decline (SCD) and related health conditions of the dynamic Bayesian network (DBN). Total number of participants: 8960. Time points: t=0 represents the initial step, in which evidence based on gender, age, education, fruit and vegetable consumption, smoking, alcohol consumption, leisure time physical activity (LTPA), BMI, and insomnia symptoms is set in the DBN model; t=0 to t=4 represent outcomes at subsequent time steps, each 5 years apart.

## Abbreviations

AI: artificial intelligence
AUC: area under the curve
AUROC: area under the receiver operating characteristic curve
BIC: Bayesian information criterion
CPT: conditional probability table
DBN: dynamic Bayesian network
HHS: Helsinki Health Study
LTPA: leisure time physical activity
MET: metabolic equivalent
OR: odds ratio
qNML: quotient normalized maximum likelihood
RCT: randomized controlled trial
ROC: receiver operating characteristic curve
SCD: subjective cognitive decline

## Data Availability

The Helsinki Health Study (HHS) survey data cannot be shared due to strict data protection laws and regulations. More information is available at the project website [54], in our data protection statement [55], and from the HHS research group [56]. Permission for the secondary use of health and social data was granted by the Finnish Social and Health Data Permit Authority, Findata.
