# Supplementary material for: Forecasting Subjective Cognitive Decline: AI Approach Using Dynamic Bayesian Networks
Source: J Med Internet Res. 2025 May 6;27:e65028. doi: 10.2196/65028 (PMC12093071; doi:10.2196/65028)
Supplement: Multimedia Appendix 1 [file jmir_v27i1e65028_app1.pdf]

Methods Appendix 2:
Validation of the subjective cognitive decline (SCD) instrument

List of Appendix Figure and Tables

Table S1. Subjective cognitive decline (SCD)* by consent given by the participants of the Helsinki Health Study in 2017 (Phase 4, N=6832, age 55–77 years) and 2022 (Phase 5, N=5950, age 60–82 years), with linkage consent referring to permission to link survey data with register data.

Figure S1. The receiver operating characteristic (ROC) curve: comparing dementia diagnoses or dementia medication purchases derived from the register data and subjective cognitive decline (SCD) among participants with consent to link their survey data with register data. Area under the curve (AUC) for Phase 4: 0.78 (N=5175) and for Phase 5: 0.75 (N=4560).

Table S2. Percentages of subjective cognitive decline (SCD)* among the participants of the Helsinki Health Study in 2017 (Phase 4, N=6832, age 55-77 years) and 2022 (Phase 5, N=5950, age 60–82 years).

Table S3. Pearson's cross-correlation for domains of subjective cognitive decline (SCD)

Table S4. Prevalence of dementia according to subjective cognitive decline (SCD) among the participants of the Helsinki Health Study in 2017 (Phase 4, N=6832, age 55–77 years) and 2022 (Phase 5, N=5950, age 60–82 years), with consent to link their survey data with register data. In the Care Register for Health Care (inpatient care) the prevalence of Alzheimer's disease was 65%.

Table S5. Register data on dementia and subjective cognitive decline (SCD) among the participants of the Helsinki Health Study in 2017 (Phase 4, N=6832, age 55–77 years) and 2022 (Phase 5, N=5950, age 60–82 years), with consent to link their survey data with register data. Age-adjusted binomial regression analysis (prevalence ratios and 95% confidence intervals)

Table S6. Subjective cognitive decline (memory, learning, concentration) among the participants of the Helsinki Health Study in 2017 (Phase 4, N=6832, age 55–77 years) and 2022 (Phase 5, N=5950, age 60–82 years). Age-adjusted binomial regression analysis (prevalence ratios and their 95% confidence intervals)

Table S7. Health functioning (RAND-36). Mean scores and standard deviations (SD) of subjective cognitive decline (SCD) among the participants of the Helsinki Health Study in 2017 (Phase 4, N=6832, age 55–77 years) and 2022 (Phase 5, N=5950, age 60–82 years)

We tested the subjective cognitive decline (SCD) measure [1] against register-based data regarding International Classification of Diseases (ICD-10) codes [2] for dementia, and dementia medications based on the Anatomical Therapeutic Chemical (ATC-codes) classification [3]. In Phase 4, 76%, and in Phase 5, 88% of the participants in our final analytic sample gave informed consent for linking their survey responses with register data. Register data were obtained from the Social Insurance Institution of Finland,[4] the Care Register for Health Care (inpatient care) [5] and the Register of Primary Health Care Visits [6]. The final analytic sample for the validation study comprised 5175 individuals in Phase 4 and 4560 individuals in Phase 5 with consent to register data linkage [7]. The data were analyzed following national data protection laws in Kapseli, which is a secure operating environment for analyzing sensitive individual level data under the Act on the Secondary Use of Health and Social Data [8]. Participants consenting and non-consenting to register data linkage were broadly similar but age, occupational class, and sickness absence affected somewhat to giving consent [9]. Regarding SCD, there were no differences between those consenting and not consenting to register data linkage **(Table S1)**.

From the register of the Social Insurance Institution of Finland, we used data on reimbursed dementia medication (ATC codes N06D) purchases 2000–2022 [3]. For diagnostic codes (ICD-10) from 1998–2022 in the Care Register for Health Care (inpatient care and outpatient visits in specialized health care) and from 2011–2022 in the Register of Primary Health Care Visits, we used the following codes: Alzheimer's disease (F00, G30.0, G30.1, G30.8, G30.9), vascular dementia (F01), and other types of dementia (F02, F03, G21.3, G13.8, G31.0, G31.1, G31.2, G31.8, G21.9, G91.2, F10.73, F11.73, F14.72, F16.73, F18.73, F19.73) [2].

Data from all the register sources were merged for examining how the register data on dementia matches with SCD self-reported in surveys. Measure for dementia was categorized as follows: "No," "Yes, in 2017 (Phase 4)," and "Yes, after 2017 (Phase 5)".

The prevalence of SCD was examined across the domains of memory, learning, and concentration and as a total score.

The prevalence of dementia by SCD was calculated first. Next age-adjusted binomial regression models were fitted to calculate prevalence ratios (PRs) and their 95% confidence intervals (95% CIs) to examine differences between SCD and register based dementia. Finally, receiver operating characteristic (ROC) curve analyses were conducted to test the diagnostic performance of SCD in relation to the register data [10].

In addition, the prevalence and prevalence ratios of SCD against depression, pain status, physical activity, diabetes, high serum cholesterol, and hypertension were calculated. Health functioning by the RAND-36 health survey [11] results were analyzed by comparing the mean scores between individuals who reported that their memory, learning, and concentration were good, and those who reported SCD.

Table S1. Subjective cognitive decline (SCD)[a] by consent given by the participants of the Helsinki Health Study in 2017 (Phase 4, N=6832, age 55–77 years) and 2022 (Phase 5, N=5950, age 60–82 years), with consent referring to permission to link survey data with register data.

Phase 4

**Consent**	**Good**	**Declined**	**Total**	**P value**
Participants (N)	3173	3502	6675	
Not available	43.2%	56.8%	100.0%	0.097
Yes	47.6%	52.4%	100.0%	
No	49.0%	51.0%	100.0%	
Total	47.5%	52.5%	100.0%	

Phase 5

**Consent**	**Good**	**Declined**	**Total**	**P value**
Participants (N)	2763	3067	5830	
Not available	44.7%	55.3%	100.0%	0.21
Yes	48.0%	52.0%	100.0%	
No	45.6%	54.4%	100.0%	
Total	47.4%	52.6%	100.0%	

[a] The instrument is derived from the TOIMIA database which is maintained by the Finnish Institute for Health and Welfare.[1]

Figure S1. The receiver operating characteristic (ROC) curve: comparing dementia diagnoses or dementia medication purchases derived from the register data and subjective cognitive decline (SCD) among participants with consent to link their survey data with register data. Area under the curve (AUC) for Phase 4: 0.78 (N=5175) and for Phase 5: 0.75 (N=4560).

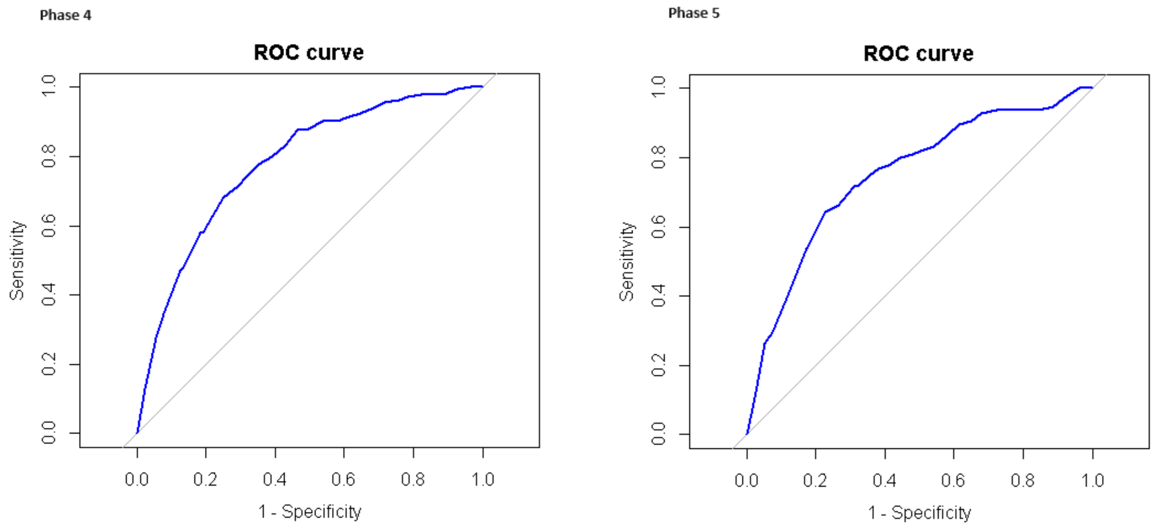

Table S2. Percentages of subjective cognitive decline (SCD)[a] among the participants of the Helsinki Health Study in 2017 (Phase 4, N=6832, age 55-77 years) and 2022 (Phase 5, N=5950, age 60–82 years).

	**Memory** ***(N=6762)***	**Learning** ***(N=6714)***	**Concentration** ***(N=6718)***
Very well	12.6%	6.7%	13.0%
Well	54.9%	45.6%	56.5%
Satisfactorily	29.2%	41.3%	27.2%
Poorly	3.1%	5.9%	3.0%
Very poorly	0.2%	0.5%	0.4%
Total	100.0%	100.0%	100.0%

**Phase 5**	**Memory** ***(N=5865)***	**Learning** ***(N=5879)***	**Concentration** ***(N=5888)***
Very well	16.1%	8.9%	16.5%
Well	52.5%	43.2%	52.4%
Satisfactorily	28.0%	40.7%	26.7%
Poorly	3.0%	6.4%	3.8%
Very poorly	0.4%	0.8%	0.6%
Total	100.0%	100.0%	100.0%

[a] The instrument is derived from the TOIMIA database which is maintained by the Finnish Institute for Health and Welfare.[1]

Table S3: Pearson's cross-correlation for domains of subjective cognitive decline (SCD)[a]

Phase 4	Memory	Learning	Concentration
**Memory**	1.00	0.59	0.58
**Learning**	0.59	1.00	0.57
**Concentration**	0.58	0.57	1.00
**Phase5**	**Memory**	**Learning**	**Concentration**
**Memory**	1.00	0.60	0.53
**Learning**	0.60	1.00	0.56
**Concentration**	0.53	0.56	1.00

[a]The instrument is derived from the TOIMIA database which is maintained by the Finnish Institute for Health and Welfare.[1]

Table S4. Prevalence of dementia according to subjective cognitive decline (SCD)[a] among the participants of the Helsinki Health Study in 2017 (Phase 4, N=6832, age 55–77 years) and 2022 (Phase 5, N=5950, age 60–82 years), with consent to link their survey data with register data. In the Care Register for Health Care (inpatient care) the prevalence of Alzheimer's disease was 65%.

	Phase 4 (2017)				Phase 5 (2022)			
**Memory**	**Good**	**Declined**	**Total**	**P value**	**Good**	**Declined**	**Total**	**P value**
Participants (N)	3524	1634	5158		3125	1401	4526	
With dementia (%)								
No	97.9	92.7	96.3	<0.001	99.1	94.8	97.8	<0.001
Yes in 2017	0.6	2.0	1.0		0.9	5.2	2.2	
Yes after 2017	1.5	5.3	2.7		0.0	0.0	0.0	
Total	100	100	100		100	100	100	
**Learning**	**Good**	**Declined**	**Total**		**Good**	**Declined**	**Total**	
Participants (N)	2674	2449	5123		2387	2155	4542	
With dementia (%)								
No	98.1	94.4	96.3	<0.001	99.1	96.3	97.8	<0.001
Yes in 2017	0.5	1.6	1.0		0.9	3.7	2.2	
Yes after 2017	1.4	4.0	2.6		0.0	0.0	0.0	
Total	100	100	100		100	100	100	
**Concentration**	**Good**	**Declined**	**Total**		**Good**	**Declined**	**Total**	
Participants (N)	3594	1530	5124		3170	1377	4547	
With dementia (%)								
No	97.5	93.5	96.3	<0.001	98.6	96.2	97.8	<0.001
Yes in 2017	0.7	1.9	1.1		1.4	3.8	2.2	
Yes after 2017	1.8	4.6	2.7		0.0	0.0	0.0	
Total	100	100	100		100	100	100	

P value from the chi-squared test

[a] The instrument is derived from the TOIMIA database which is maintained by the Finnish Institute for Health and Welfare.[1]

Table S5. Register data on dementia and subjective cognitive decline (SCD)[a] among the participants of the Helsinki Health Study in 2017 (Phase 4, N=6832, age 55–77 years) and 2022 (Phase 5, N=5950, age 60–82 years), with consent to link their survey data with register data. Age-adjusted binomial regression analysis (prevalence ratios and 95% confidence intervals).

**Dementia**	**Phase 4 (2017)**			**Phase 5 (2022)**		
	**PR**	**95% CI**	**AUC**	**PR**	**95% CI**	**AUC**
Age (continuous)	1.17	(1.14, 1.20)		1.15	(1.11, 1.19)	
Sex (ref Men)						
Women	1.08	(0.78, 1.52)		0.89	(0.58, 1.42)	
Education (ref Basic)						
Secondary	0.94	(0.67, 1.32)		0.82	(0.50, 1.32)	
Higher	0.89	(0.64, 1.22)		0.86	(0.55, 1.35)	
Subjective cognitive decline (ref good)	2.38	(1.69, 3.43)	0.78	2.90	(1.77, 5.01)	0.75
Declined memory (ref good)	2.71	(2.04, 3.62)	0.79	4.32	(2.82, 6.82)	0.78
Declined learning (ref good)	1.96	(1.43, 2.73)	0.77	2.95	(1.85, 4.92)	0.76
Declined concentration(ref good)	1.99	(1.51, 2.63)	0.77	2.14	(1.44, 3.19)	0.74

PR, prevalance ratio

CI, confidence interval

AUC, Area under the curve

[a] The instrument is derived from the TOIMIA database which is maintained by the Finnish Institute for Health and Welfare.[1]

Table S6. Subjective cognitive decline (memory, learning, concentration)[a] among the participants of the Helsinki Health Study in 2017 (Phase 4, N=6832, age 55–77 years) and 2022 (Phase 5, N=5950, age 60–82 years). Age-adjusted binomial regression analysis (prevalence ratios and their 95% confidence intervals)

	Phase 4 (2017) all	Phase 5 (2022) all	Phase 4 (2017) both	Phase 5 (2022) both	Phase 4 (2017) only
**SOCIODEMOGRAPHIC FACTORS**					
**Age** (continuous)	1.06 (1.05,1.07)	1.07 (1.06,1.08)	1.05 (1.05,1.06)	1.07 (1.06,1.08)	1.07 (1.05,1.09)
**Gender**					
Men	1.35 (1.19,1.53)	1.25 (1.08,1.44)	1.38 (1.19,1.59)	1.25 (1.08,1.45)	1.26 (0.96,1.65)
Women (ref)					
**Education**					
Basic	1.60 (1.41,1.80)	1.68 (1.46,1.92)	1.66 (1.45,1.90)	1.68 (1.46,1.93)	1.36 (1.02,1.82)
Secondary	1.30 (1.15,1.48)	1.26 (1.10,1.45)	1.32 (1.15,1.52)	1.24 (1.08,1.43)	1.17 (0.85,1.61)
Higher (ref)					
**RISK FACTORS**					
**Smoking**					
Never (ref)					
Ex-smoker	1.07 (0.96,1.20)	1.10 (0.98,1.25)	1.04 (0.91,1.17)	1.10 (0.97,1.25)	1.31 (0.98,1.74)
Current	1.23 (1.05,1.45)	1.12 (0.91,1.38)	1.21 (1.00,1.47)	1.14 (0.92,1.41)	1.41 (1.01,1.97)
**Alcohol consumption**					
No consumption	1.21 (1.04,1.39)	1.25 (1.08,1.45)	1.18 (1.00,1.40)	1.25 (1.08,1.45)	1.30 (0.97,1.74)

Moderate consumption (ref)					
High consumption	1.12 (0.93,1.35)	1.03 (0.81,1.31)	1.11 (0.91,1.36)	1.02 (0.80,1.31)	1.16 (0.77,1.77)
High risk consumption	1.51 (1.22,1.87)	1.33 (1.01,1.77)	1.45 (1.14,1.85)	1.33 (1.01,1.77)	1.74 (1.11,2.76)
**Physical activity**					
Physically inactive(MET < 14)	1.51 (1.34,1.69)	1.58 (1.38,1.80)	1.43 (1.25,1.64)	1.60 (1.40,1.83)	1.79 (1.40,2.30)
Physically active(MET > 14) (ref)					
**Fruit and vegetable consumption**					
Non-daily consumer	1.59 (1.40,1.81)	1.57 (1.33,1.85)	1.60 (1.38,1.85)	1.58 (1.35,1.87)	1.57 (1.20,2.06)
Daily consumer of either	1.34 (1.19,1.51)	1.36 (1.19,1.55)	1.36 (1.19,1.56)	1.35 (1.18,1.55)	1.25 (0.95,1.64)
Daily consumer both (ref)					
**Insomnia symptoms**					
<4 nights/month (ref)					
4-14nights/month	1.93 (1.70,2.19)	1.64 (1.43,1.88)	1.91 (1.66,2.20)	1.65 (1.43,1.89)	2.02 (1.50,2.73)
>14 nights/month	3.20 (2.80,3.66)	2.51 (2.17,2.91)	3.12 (2.69,3.62)	2.52 (2.18,2.93)	3.59 (2.62,4.94)
**Body mass index**					
Underweight -18.5	1.16 (0.73,1.86)	0.89 (0.53,1.49)	0.94 (0.53,1.68)	0.90 (0.54,1.50)	1.61 (0.70,4.03)
Recommended healthy weight 18.5+ (ref)					
Overweight 25+	1.02 (0.91,1.14)	1.12 (0.98,1.27)	1.10 (0.97,1.25)	1.12 (0.99,1.28)	0.74 (0.58,0.96)
Obesity +30	1.05 (0.92,1.19)	1.18 (1.02,1.37)	1.04 (0.90,1.20)	1.20 (1.03,1.39)	1.06 (0.79,1.41)

HEALTH FACTORS AND PHYSICIAN DIAGNOSED DISEASES

**Pain**	1.70 (1.53,1.88)	1.69 (1.51,1.90)	1.65 (1.47,1.85)	1.71 (1.53,1.92)	1.93 (1.52,2.44)
**Depression**	2.70 (2.28,3.20)	2.87 (2.37,3.50)	2.56 (2.13,3.09)	2.89 (2.38,3.52)	3.34 (2.30,4.92)
**Diabetes**	1.36 (1.16,1.60)	1.34 (1.13,1.58)	1.28 (1.07,1.54)	1.34 (1.13,1.58)	1.65 (1.18,2.32)
**Hypertension**	1.33 (1.19,1.49)	1.24 (1.10,1.40)	1.32 (1.17,1.50)	1.24 (1.10,1.40)	1.38 (1.07,1.78)
**High cholesterol**	1.49 (1.33,1.67)	1.34 (1.18,1.52)	1.51 (1.33,1.72)	1.34 (1.18,1.52)	1.40 (1.07,1.82)

PR, prevalance ratio

CI, confidence interval

[a] The instrument is derived from the TOIMIA database which is maintained by the Finnish Institute for Health and Welfare.[1]

Table S7. Health functioning (RAND-36) [11]. Mean score and standard deviation (SD) of subjective cognitive decline (SCD) among the participants of the Helsinki Health Study in 2017 (Phase 4, N=6832, age 55–77 years) and 2022 (Phase 5, N=5950, age 60–82 years)

**Health-related quality of life**		**Phase 4 all**	**Phase 5 all**	**Phase 4 both 4 & 5**	**Phase 5 both 4 & 5**	**Phase 4 not in 5**
**Physical functioning**						
SCD	Good	85.3 (18.2)	83.5 (19.4)	86.1 (17.3)	83.6 (19.2)	81.9 (21.3
	Declined	76.8 (21.2)	71.9 (23.8)	78.4 (19.9)	72.2 (23.4)	70.5 (24.7)
Changes in SCD	Stable good			86.9 (16.9)	84.2 (18.9)	
	Improved			83.0 (18.1)	81.4 (20.0)	
	Declined			83.3 (18.5)	75.5 (23.4)	
	Stable poor			77.4 (20.1)	71.3 (23.3)	
**Role functioning/physical**						
SCD	Good	81.4 (32.3)	79.6 (33.6)	82.8 (30.7)	79.9 (33.4)	75.4 (37.6)
	Declined	62.3 (39.9)	55.2 (41.3)	64.9 (38.8)	55.5 (41.1)	52.2 (42.4)
Changes in SCD	Stable good			85.1 (28.9)	81.7 (32.0)	
	Improved			71.1 (36.5)	73.2 (37.4)	
	Declined			74.9 (35.3)	64.5 (39.6)	
	Stable poor			63.4 (39.2)	53.2 (41.1)	
**Social functioning**						
SCD	Good	90.6 (17.0)	88.9 (18.8)	91.1 (16.4)	89.0 (18.7)	88.8 (19.0)
	Declined	79.7 (23.5)	76.1 (25.2)	80.9 (23.0)	76.5 (24.9)	75.0 (25.1)
Changes in SCD	Stable good			91.8 (16.0)	89.9 (17.9)	
	Improved			84.0 (21.0)	85.3 (21.2)	
	Declined			88.7 (17.6)	81.0 (23.5)	
	Stable poor			80.2 (23.4)	75.3 (25.1)	

**Bodily pain**						
SCD	Good	75.3 (21.6)	74.6 (21.4)	75.5 (21.2)	74.6 (21.3)	74.3 (23.5
	Declined	66.7 (23.1)	64.8 (23.7)	67.5 (22.5)	65.0 (23.4)	63.4 (24.8)
Changes in SCD	Stable good			76.6 (21.0)	75.4 (21.1)	
	Improved			70.8 (21.8)	71.7 (21.8)	
	Declined			71.4 (21.3)	69.2 (23.3)	
	Stable poor			66.8 (22.6)	64.0 (23.3)	
**Emotional well-being**						
SCD	Good	84.7 (12.7)	83.7 (13.1)	84.9 (12.3)	83.7 (13.1)	83.6 (13.9)
	Declined	74.8 (16.8)	72.7 (17.6)	75.6 (16.3)	73.0 (17.3)	71.9 (18.2)
Changes in SCD	Stable good			85.7 (12.0)	84.7 (12.4)	
	Improved			77.4 (15.3)	79.9 (14.7)	
	Declined			82.1 (13.1)	76.8 (15.8)	
	Stable poor			75.1 (16.5)	72.0 (17.6)	
**(Role functioning/emotional)**						
SCD	Good	89.0 (26.0)	86.5 (28.4)	89.8 (24.7)	86.5 (28.4)	85.3 (30.7
	Declined	69.9 (38.8)	62.4 (41.2)	72.2 (37.2)	63.0 (40.8)	60.9 (43.1)
Changes in SCD	Stable good			91.0 (23.4)	88.0 (26.9)	
	Improved			77.5 (35.2)	81.0 (32.9)	
	Declined			85.8 (28.2)	72.0 (38.5)	
	Stable poor			70.9 (37.6)	60.7 (41.1)	
**Energy**						
SCD	Good	71.6 (16.6)	70.0 (16.9)	71.7 (16.3)	70.0 (16.8)	71.0 (17.7)
	Declined	57.6 (19.4)	54.8 (19.8)	58.3 (19.1)	55.1 (19.5)	55.1 (20.5)
Changes in SCD	Stable good			72.9 (16.2)	71.3 (16.5)	
	Improved			60.8 (17.8)	65.2 (17.3)	
	Declined			67.9 (16.1)	59.1 (19.1)	
	Stable poor			57.7 (19.3)	54.0 (19.4)	

**General health perceptions**						
SCD	Good	68.0 (18.5)	66.6 (18.4)	68.6 (18.3)	66.6 (18.3)	65.5 (19.1)
	Declined	54.7 (19.3)	52.5 (18.7)	56.1 (18.9)	52.7 (18.6)	49.7 (19.9)
Changes in SCD	Stable good			70.0 (18.2)	67.7 (18.0)	
	Improved			60.5 (18.8)	62.8 (18.8)	
	Declined			63.5 (17.7)	57.9 (19.6)	
	Stable poor			55.0 (18.7)	51.4 (18.2)	

References

1. The Finnish Institute for Health and Welfare, (THL). TOIMIA-tietokanta. 2011 Jan 17 [cited 2024 Jun 15]; Available from: https://www.terveysportti.fi/apps/dtk/tmi/article/tmm00047

2. World Health Organization. ICD-10 : international statistical classification of diseases and related health problems : tenth revision. 2nd ed. World Health Organization; 2004. p. Spanish version, 1st edition published by PAHO as Publicación Científica 544.

3. WHO Collaborating Centre for Drug Statistics Methodology. International Language for Drug Utilisation Research [Internet]. 2024 [cited 2024 Jun 15]. Available from: https://www.whocc.no/

4. Finland (Kela) TSII of. Kela's Medication Prescriptions Registry [Internet]. 2024 [cited 2024 Jun 15]. Available from: https://www.kela.fi/main-page

5. Health FI for, Welfare. Care Register for Health Care (Hilmo) [Internet]. 2024 [cited 2024 Jun 15]. Available from: https://thl.fi/en/statistics-and-data/data-and-services/register-descriptions/care-register-for-health-care

6. Health FI for, Welfare. Register of Primary Health Care Visits (Avohilmo) [Internet]. 2024 [cited 2024 Jun 15]. Available from: https://thl.fi/en/statistics-and-data/data-and-services/register-descriptions/register-of-primary-health-care-visits

7. Lahelma E, Aittomäki A, Laaksonen M, Lallukka T, Martikainen P, Piha K, et al. Cohort Profile: The Helsinki Health Study. International Journal of Epidemiology. 2013 Jun;42(3):722–30.

8. Ministry of Social Affairs and Health. Secondary Use of Health and Social Data [Internet]. 2024 [cited 2024 Jun 15]. Available from: https://stm.fi/en/secondary-use-of-health-and-social-data

9. Laaksonen M, Aittomäki A, Lallukka T, Rahkonen O, Saastamoinen P, Silventoinen K, et al. Register-based study among employees showed small nonparticipation bias in health surveys and check-ups. Journal of Clinical Epidemiology. 2008 Sep;61(9):900–6.

10. Zweig MH, Campbell G. Receiver-operating characteristic (ROC) plots: a fundamental evaluation tool in clinical medicine. Clinical Chemistry. 1993 Apr 1;39(4):561–77.

11. Hays RD, Sherbourne CD, Mazel RM. The rand 36-item health survey 1.0. Health Economics. 1993 Oct;2(3):217–27.
