# Supplementary material for: Forecasting Subjective Cognitive Decline: AI Approach Using Dynamic Bayesian Networks
Source: J Med Internet Res. 2025 May 6;27:e65028. doi: 10.2196/65028 (PMC12093071; doi:10.2196/65028)
Supplement: Multimedia Appendix 2 [file jmir_v27i1e65028_app2.docx]

**Multimedia Appendix 1.** Response rates by variable and study phase.

| Variables | | Phases | | | | |
| --- | --- | --- | --- | --- | --- | --- |
|  | | Phase 1 (2000-2002; N=8960), n (%) | Phase 2 (2007; N=7332), n (%) | Phase 3 (2012; N=6808), n (%) | Phase 4 (2017; N=6831), n (%) | Phase 5 (2022; N=5950), n (%) |
|  | |  |  |  |  |  |
| **Smoking** | | | | | | |
|  | Response | 8518 (95.1) | 6939 (94.6) | 6116 (89.8) | 5838 (85.5) | 5016 (84.3) |
|  | No answer | 442 (4.9) | 393 (5.4) | 692 (10.2) | 993 (14.5) | 934 (15.7) |
| **Alcohol consumption** | | | | | | |
|  | Response | 8573 (95.7) | 7067 (96.4) | 6548 (96.2) | 6625 (97.0) | 5671 (95.3) |
|  | No answer | 387 (4.3) | 265 (3.6) | 260 (3.8) | 206 (3.0) | 279 (4.7) |
| **LTPA^a^** | | | | | | |
|  | Response | 8869 (99.0) | 7256 (99.0) | 6711 (98.6) | 6719 (98.4) | 5896 (99.1) |
|  | No answer | 91 (1.0) | 76 (1.0) | 97 (1.4) | 112 (1.6) | 54 (0.9) |
| **Fruit and vegetable consumption** | | | | | | |
|  | Response | 8873 (99.1) | 7203 (98.4) | 6675 (98.0) | 6635 (97.5) | 5811 (97.7) |
|  | No answer | 87 (0.9) | 129 (1.6) | 133 (2.0) | 173 (2.5) | 139 (2.3) |
| **BMI** | | | | | | |
|  | Response | 8853 (98.8) | 7252 (98.9) | 6706 (98.5) | 6743 (98.7) | 5863 (98.5) |
|  | No answer | 107 (1.2) | 80 (1.1) | 102 (1.5) | 88 (1.3) | 87 (1.5) |
| **Insomnia symptoms (nights/month)** | | | | | | |
|  | Response | 8216 (91.7) | 6719 (91.6) | 6189 (90.9) | 5855 (85.7) | 5260 (88.4) |
|  | No answer | 744 (8.3) | 613 (8.4) | 619 (9.1) | 976 (14.3) | 690 (11.6) |
| **Memory** | | | | | | |
|  | Response | —^b^ | — | — | 6762 (99.0) | 5865 (98.6) |
|  | No answer | — | — | — | 69 (1.0) | 85 (1.4) |
| **Learning** | | | | | | |
|  | Response | — | — | — | 6714 (98.3) | 5879 (98.8) |
|  | No answer | — | — | — | 117 (1.7) | 71 (1.2) |
| **Concentration** | | | | | | |
|  | Response | — | — | — | 6718 (98.3) | 5888 (98.9) |
|  | No answer | — | — | — | 113 (1.7) | 62 (1.1) |
| **Pain** | | | | | | |
|  | Response | 8755 (97.7) | 7106 (96.9) | 6648 (97.6) | 6494 (95.1) | 5711 (96.0) |
|  | No answer | 205 (2.3) | 226 (3.1) | 160 (2.4) | 337 (4.9) | 239 (4.0) |
| **Hypertension** | | | | | | |
|  | Response | 8865 (98.9) | 7207 (98.3) | 6699 (98.4) | 5431 (79.5) | 5017 (84.3) |
|  | No answer | 95 (1.1) | 125 (1.7) | 109 (1.6) | 1400 (20.5) | 933 (15.7) |
| **High cholesterol** | | | | | | |
|  | Response | 8874 (99.0) | 7252 (98.9) | 6773 (99.5) | 5067 (74.2) | 4640 (78.0) |
|  | No answer | 86 (1.0) | 80 (1.1) | 35 (0.5) | 1764 (25.8) | 1310 (22.0) |
| **Diabetes** | | | | | | |
|  | Response | 7889 (88.0) | 5680 (77.5) | 5344 (78.5) | 4658 (68.2) | 4061 (68.3) |
|  | No answer | 1071 (12.0) | 1652 (22.5) | 1464 (21.5) | 2173 (31.8) | 1889 (31.7) |
| **Mental disorders** | | | | | | |
|  | Response | 8102 (90.5) | 5897 (80.4) | 5443 (80.0) | 4638 (67.9) | 4056 (68.2) |
|  | No answer | 858 (9.5) | 1435 (19.6) | 1365 (20.0) | 2193 (32.1) | 1894 (31.8) |

^a^LTPA: leisure time physical activity.

^b^Not applicable.
